# iPSC-Derived Striatal Medium Spiny Neurons from Patients with Multiple System Atrophy Show Hypoexcitability and Elevated α-Synuclein Release

**DOI:** 10.3390/cells12020223

**Published:** 2023-01-04

**Authors:** Lisa M. Henkel, Svenja Kankowski, Thiemo M. Moellenkamp, Nadine J. Smandzich, Sigrid Schwarz, Alessio Di Fonzo, Gudrun Göhring, Günter Höglinger, Florian Wegner

**Affiliations:** 1 Department of Neurology, Hannover Medical School, Carl-Neuberg-Str. 1, 30625 Hannover, Germany; 2 Center for Systems Neuroscience, Bünteweg 2, 30559 Hannover, Germany; 3 Institute of Neuroanatomy and Cell Biology, Hannover Medical School, 30625 Hanover, Germany; 4 Curiositas-ad-sanum Studien und Beratungs GmbH, Krankenhausstr. 3, 83527 Haag in Oberbayern, Germany; 5 Foundation IRCCS Ca’ Granda Hospital Maggiore Policlinico, Unità di Neurologia, 20122 Milan, Italy; 6 Department of Human Genetics, Hannover Medical School, Carl-Neuberg-Str. 1, 30625 Hannover, Germany; 7 Department of Neurology, University Hospital of Munich, Marchioninistr. 15, 81377 Munich, Germany

**Keywords:** induced pluripotent stem cells (iPSCs), striatal medium spiny neurons (MSNs), multiple system atrophy (MSA), α-synuclein, hypoexcitability

## Abstract

Multiple system atrophy of the parkinsonian type (MSA-P) is a rare, fatal neurodegenerative disease with sporadic onset. It is still unknown if MSA-P is a primary oligodendropathy or caused by neuronal pathophysiology leading to severe, α-synuclein-associated neurodegeneration, mainly in the striatum. In this study, we generated and differentiated induced pluripotent stem cells (iPSCs) from patients with the clinical diagnosis of probable MSA-P (*n* = 3) and from three matched healthy controls into GABAergic striatal medium spiny neurons (MSNs). We found a significantly elevated release and neuronal distribution for α-synuclein, as well as hypoexcitability in the MSNs derived from the MSA-P patients compared to the healthy controls. These data suggest that the striatal hypoexcitable neurons of MSA-P patients contribute to a pathological α-synuclein burden which is likely to spread to neighboring cells and projection targets, facilitating disease progression.

## 1. Introduction

Multiple system atrophy (MSA) is a rare, fatal neurodegenerative disease with a sporadic onset and a still unknown cause. Patients with MSA are clinically classified into two subtypes: MSA-P with predominantly parkinsonian symptoms and striatonigral degeneration, and MSA-C with predominant cerebellar ataxia and olivopontocerebellar atrophy as morphological phenotype [1,2].

Independent of the presented MSA subtype, the formation of glial cytoplasmic inclusions (GCIs) consisting of accumulated α-synuclein aggregates presents the pathological hallmark of the disease. Together with Parkinson’s disease (PD) and dementia with Lewy bodies, MSA is classified as synucleinopathy [3]. The recurrence of GCIs in MSA brains is significantly correlated with the severity of neuronal cell loss [4]. It remains unknown if MSA is a primary oligodendropathy or if the formation of GCIs is secondary to neuronal pathophysiology [5]. Severe neuronal losses in various brain regions, such as the putamen, pons, cerebellum, inferior olive, and substantia nigra, are characteristic of MSA, while the number of oligodendrocytes seems to be preserved, despite myelin loss [6]. The underlying molecular mechanisms remain obscure. Converging evidence suggests a “prion-like” spreading of misfolded α-synuclein as a key event in the pathogenic cascade of MSA [7,8,9].

Due to the typical association of MSA-P with the degeneration of striatal GABAergic medium spiny neurons (MSNs) [10], we chose to differentiate the induced pluripotent stem cells from patients with clinically diagnosed MSA-P into MSNs, which had not been carried out before, adapting two of our previously established protocols [11,12]. The aim of this study is to analyze the disease-specific morphological and functional phenotypes of MSA-P MSNs compared to age-matched healthy controls. We characterized the morphometric features, such as GABAergic synaptic density, evaluated the functional properties with whole-cell patch-clamp recordings and calcium imaging, and analyzed the gene expression of the GABA receptors and voltage-gated Ca^2+^ channel subunits in detail. Furthermore, we investigated the distribution of α-synuclein in the MSNs, as well as its release into the surrounding medium.

## 2. Materials and Methods

### 2.1. Generation and Cultivation of Human, Induced Pluripotent Stem Cells (iPSCs)

In addition to one control iPSC line commercially available through the StemBANCC consortium (cell line SFC084-03-02-01A) and one MSA-P cell line previously established and characterized by Compagnoni et al. [13], we generated two new iPSC lines for each group (MSA-P and healthy control) at the Hannover Medical School (Table 1).

Skin punch biopsies of two MSA-P patients (probable diagnosis using Gilman criteria [1]), referred to as MSA-P2 and MSA-P3, and two healthy age- and sex-matched control persons, referred to as CTR1 and CTR3 (Table 1), were taken after written informed consent and with approval by the ethics committee of Hannover Medical School (Nr. 8666_BO_K_2019). The MSA-P patients included in this study were still able to live a mostly independent life with no need for constant professional care at the time of the biopsy. Skin biopsies were cultivated in fibroblast medium consisting of DMEM (Thermo Fisher Scientific, Waltham, MA, USA) supplemented with 1.1 µg Sodium-Pyruvate, 1% Penicillin-Streptomycin (both from Thermo Fisher Scientific, Waltham, MA, USA), 10% fetal calf serum, and 0.2 µg Uridine (both from Sigma-Aldrich, St. Louis, MO, USA), promoting the growth of human dermal fibroblasts (HDFs) but not alternative cell types like keratinocytes from the biopsies. Fibroblasts were cultured for two to three weeks with a passage once a week before reprogramming was performed using the StemRNA™ 3rd Gen Reprogramming Kit (ReproCELL, Yokohama, Kanagawa, Japan) according to manufacturer’s instructions, as previously reported [14]. Briefly, HDFs were seeded in mTeSR medium (Stemcell Technologies, Vancouver, BC, Canada) with 1% Penicillin-Streptomycin (Thermo Fisher Scientific, Waltham, MA, USA) on hESC-qualified Matrigel (Corning, Corning, NY, USA). After 24 h of seeding, HDFs were transfected for four consecutive days with immune evasion mRNAs E3, K3, and B18R (EKB), the reprogramming mRNAs OCT4, SOX2, KLF4, cMYC, NANOG, and LIN28 (OSKMNL), and miRNA using RNAiMAX and OptiMEM (both from Thermo Fisher Scientific, Waltham, MA, USA). IPSC cultivation medium was exchanged every day, and after 2 weeks, iPSC colonies were manually picked and expanded. iPSCs were passaged every 5–7 days using 0.2 mM EDTA with the addition of 10 µM Rho kinase (ROCK) inhibitor Y27632 (Stemcell Technologies, Vancouver, BC, Canada) for the first 24 h after the split to increase cell survival.

### 2.2. Trilineage Differentiation

The STEMdiff Trilineage Differentiation Kit (Stemcell Technologies, Vancouver, BC, Canada) was used according to the manufacturer’s protocol. Briefly, iPSCs were seeded on coverslips coated with hESC-qualified Matrigel in mTeSR. After 24 h, the medium was switched to the respective medium for each germ layer (Stemcell Technologies, Vancouver, BC, Canada) and cultivated for 5 d for meso- and endoderm and 7 d for ectoderm, with daily medium change. Then, cells were fixed and analyzed by immunocytochemistry.

### 2.3. Karyotyping

Karyotyping was performed by G-banding to examine the integrity of the chromosomes by the Department of Human Genetics at Hannover Medical School (MHH).

### 2.4. Differentiation into Striatal Medium Spiny Neurons (MSNs)

Striatal MSN differentiation was adapted from previously published protocols by Stanslowsky et al. [11] and Capetian et al. [12]. At day 0 of differentiation, embryoid body (EB) formation was initiated by collecting detached iPSCs using sedimentation and resuspension in mTeSR (Stemcell Technologies, Vancouver, BC, Canada) supplemented with 1 µM dorsomorphin (Tocris, Bio-Techne, Minneapolis, MN, USA), 1 µM IWP2 (Merck, Darmstadt, Germany), 10 µM ROCK inhibitor Y27632 (Stemcell Technologies, Vancouver, BC, Canada), and 10 µM SB-431542 (Tocris, Bio-Techne, Minneapolis, MN, USA). After two days, the medium was replaced with 1:1 mTeSR/N2 medium (Knockout-DMEM/F-12, 1:100 N2 supplement, Penicillin-Streptomycin, L-Glutamin, MEM nonessential amino acids (MEM NEAA) solution, and 15 mM HEPES buffer (all from Thermo Fisher Scientific, Waltham, MA, USA)) supplemented 1µM dorsomorphin, 1 µM IWP2, and 10 µM SB-431542 (day 2). On day 4, the medium was changed to N2 medium, supplemented with the same supplement concentrations as before, with the addition of 0.2 µM purmorphamine (PMA; Enzo Life Sciences, Lörrach, Germany). On days 6 and 8, the supplementation of SB-431542 and dorsomorphin was omitted, and on day 10, no supplements were added. EBs were plated on basic Matrigel (Corning, Corning, NY, USA) coated plates at day 12 ± 1 day in N2B27 medium (1:1 DMEM/F12 and Neurobasal medium containing 1:100 B27 without vitamin A, 1:200 N2, 1% Penicillin-Streptomycin, and L-Glutamin (all from Thermo Fisher Scientific, Waltham, MA, USA)) supplemented with 20 ng/µL brain-derived neurotrophic factor (BDNF; PeproTech, Cranbury, NJ, USA), 10 ng/mL glial cell-line derived neurotrophic factor (GDNF; PeproTech, Cranbury, NJ, USA), and 50 µM dibutyryl-cAMP (dbcAMP; Sigma-Aldrich, St. Louis, MO, USA). Maturation medium was changed every other day. Once the outgrowing cells reached 70–90% confluency, they were replated as clumps, using accutase (Sigma-Aldrich, St. Louis, MO, USA) to detach them onto laminin/poly-DL-ornithine hydrobromid (Thermo Fisher Scientific, Waltham, MA, USA) coated dishes in a 1:2 and 1:4 ratio. To replate the neuronal cultures as single cells, they were again detached, diluted in at least 1:10 as single cells in maturation medium supplemented with 10 µM Y27632 ROCK inhibitor (Stemcell Technologies, Vancouver, BC, Canada) and seeded onto newly laminin/poly-DL-ornithine hydrobromid (Thermo Fisher Scientific, Waltham, MA, USA) coated dishes. ROCK inhibitor was withdrawn 24 h after splitting. Fully differentiated neuronal cells were characterized after 70 days ± 3 days. A total of three to four independent differentiations for each iPSC line (three MSA-P and three control lines, Table 1) were analyzed.

### 2.5. Immunocytochemistry

The medium was aspired, and cells were fixed for 20 min with 4% paraformaldehyde (PFA, Sigma-Aldrich, St. Louis, MO, USA) before incubation with blocking solution, consisting of 1% bovine serum albumin, 5% goat serum, and 0.3% Triton X-100 (all from Sigma-Aldrich, St. Louis, MO, USA) in PBS for 1 h. Cultures were incubated overnight at 4 °C with primary antibodies (Supplementary Table S1) diluted, accordingly, in blocking solution. Appropriate secondary antibodies (Supplementary Table S2) were incubated with the cultures for 2 h at room temperature before nuclei were counterstained with 0.1% DAPI (4,6-diamidino-2-phenylindole, 10 mg/mL, Thermo Fisher Scientific, Waltham, MA, USA) supplemented in the mounting medium. The fluorescence microscope BX61, DP72 camera, and the analysis software CellF (all from Olympus, Shinjuku, Japan) were adopted for image acquisition. Cell counts were performed within four randomly selected visual fields per coverslip from at least three independent differentiations of at least two controls and three MSA-P lines. Representative images were processed using ImageJ and the bioimaging analysis platform QuPath [15], in which cells were segmented and subsequently classified.

### 2.6. Analysis of Synaptic Density and Quantitative Image Analysis

Fluorescence images were acquired with cellSens imaging software using an Olympus BX60 Microscope equipped with an Olympus XM10 cooled CCD monochrome camera, an Olympus U-RFL-T power supply, and a 40× and 100× objective lens (all from Olympus, Shinjuku, Japan). Comparability of the quantitative parameters between the images was ensured, applying a constant exposure time, gain, and threshold for every fluorescence stain.

The density of the GABAergic synapses was quantified, as previously described [16,17]. Cells were stained with primary antibodies against β-tubulin III (Abcam, Cambridge, MA, USA) and GABA (Sigma-Aldrich, St. Louis, MO, USA). One neurite per cell was traced with a distance of 30 µm to the soma for a total length of at least 50 µm using the ImageJ plugin NeuronJ (version 1.4.3) [18]. The tracing was implemented to the ImageJ plugin SynapCountJ (version 2.0) [19] and used to detect the GABA-positive spots along the segment of the selected neurite.

Fluorescence intensity of α-synuclein clusters, stained with a primary antibody against α-synuclein (Santa Cruz Biotechnology, Dallas, TX, USA), was quantified using the ROI manager tool of ImageJ. Two regions of interest were defined: the soma and neurites of the neuron compared to the cell nucleus, which were traced according to DAPI staining. GABA(+) neurons were qualified according to GABA staining.

### 2.7. Quantification of α-Synuclein Release by Dot Blot

Conditioned medium from three independent differentiations of two control and two MSA-P (P1, P3) iPSC cell lines was collected, each at 40 and 70 ± 4 days of differentiation into MSNs. To remove cell debris, samples were spun at 1000× *g* for 10 min. Using vacuum dot blot equipment, the proteins were transferred on 0.2 µm PVDF membranes (Bio-Rad Laboratories). After blocking the membranes for 2 h at room temperature with 30% RotiBlock solution (Carl Roth, Karlsruhe, Germany), primary antibodies (Supplementary Table S1) were diluted accordingly and incubated with the membranes overnight at 4 °C. Membranes were washed (Tris-buffered saline (TBS) supplemented with 0.05% Tween-20 (Sigma-Aldrich, St. Louis, MO, USA)), and the appropriate HRP-coupled secondary antibodies (Supplementary Table S2) were incubated for 1 h at room temperature. For visualization, the membranes were incubated in Clarity Western ECL Substrate (Bio-Rad Laboratories, Hercules, CA, USA), and the images were acquired using the Odyssey Fc (LICOR Biotechnology, Lincoln, NE) imaging system. Image Studio™ Software (version 5.2.5, LI-COR Biosciences GmbH, Bad Homburg vor der Höhe, Germany) was used for signal intensity annotation, and α-synuclein signals were normalized against GAPDH and β-actin signals.

### 2.8. Quantitative Real-Time PCR

Total RNA from iPSCs and MSNs at 70 ± 3 days was extracted using the RNeasy Micro Kit (Qiagen, Venlo, The Netherlands), including a column DNase digestion, according to manufacturer’s instructions. A total of 1 µg of RNA for each reaction was transcribed into cDNA using QuantiTec reverse transcription kit (Qiagen, Venlo, The Netherlands). Quantitative real-time PCR reaction analysis was performed with 7 ng total RNA, Power SYBR-Green PCR Master Mix (Thermo Fisher Scientific, Waltham, MA, USA), and 1.75 µM forward and reverse primer. A StepOnePlus cycler (Thermo Fisher Scientific, Waltham, MA, USA) under the conditions: 95 °C for 10 min, followed by 40 cycles of 95 °C for 15 s, and 60°C for 1 min was used for the reaction analysis. Threshold cycle (Ct) values of the targets were normalized against endogene references (β-actin, β2-microglobulin (B2M), and glyceraldehyde 3-phosphate dehydrogenase (GAPDH)) for the quantification of target gene expression (Ct (target) − Ct (reference) = ΔCt). The ΔCt values of three independent differentiations per cell line, each gene in triplicate, were plotted as relative gene expression levels and illustrated as means ± standard error of the mean (SEM). To ensure the specificity of PCR products, a melting curve analysis, as recommended by the MIQUE-guidelines [20], and a serial cDNA dilution to validate the equivalent PCR efficiency of all primer pairs, were performed. Primer information is provided in Supplementary Table S3.

### 2.9. Calcium Imaging

The recording of cytosolic calcium signals in MSNs was carried out, as reported previously [11,16,21], using the fluorescent indicator Fura 2-AM (Sigma-Aldrich, St. Louis, MO, USA). In brief, neurons were visualized by an upright microscope Axioskop 2 FS plus (Carl Zeiss MicroImaging GmbH, Göttingen, Germany) connected to a Till Vision Imaging System (TILL Photonics, Gräfelfing, Germany) and a charge-coupled device (CCD) camera. The MSNs of two to four independent differentiations (70 ± 4 days) per cell line were incubated for 20 min at 37 °C with Fura 2-AM in a standard bath solution containing 140 mM NaCl, 5 mM KCl, 2 mM CaCl_2_, 10 mM glucose, and 10 mM HEPES, adjusted to pH 7.4 with NaOH. Intracellular Ca^2+^ was monitored by exciting Fura 2-AM at wavelengths of 340/380 nm, with its emission recorded every 300 ms at 510 nm. The MSNs with a preferably multipolar morphology were imaged for 6 min to measure spontaneous intracellular Ca^2+^ signal changes. As described recently [16,22], the neurotransmitters glycine, acetylcholine, GABA (all 100 µM), and glutamate (50 µM) were applied after 3–6 min baseline conditions, and the induced intracellular Ca^2+^ peaks were recorded for 1 min. Note, glycine- and GABA-evoked Ca^2+^ amplitudes suggest a minor depolarizing effect of these neurotransmitters in some cells, probably because of a high intracellular chloride concentration [23,24]. At the end of the recordings, KCl (50 mM) was applied to elicit neuronal depolarizations indicating the viability and excitability of the MSNs. After background subtraction, the 340/380 nm excitation ratio for Fura 2-AM was determined, which increases as a function of the cytosolic free Ca^2+^ concentration. Only Ca^2+^ transients with a 340/380 nm excitation ratio of Fura 2-AM ≥ 0.05 from individual neurons were analyzed to avoid noise artifacts [16]. At least two coverslips with 60–180 neurons per cell line and a minimum of two independent MSN differentiations were evaluated.

### 2.10. Electrophysiology

Whole-cell patch-clamp recordings of MSNs were carried out after 70 ± 3 days of at least three independent differentiations per cell line, as reported recently [21]. Briefly, patch pipettes had resistances of 3–4 MΩ after filling with an internal solution that consisted of 153 mM KCl, 1 mM MgCl_2_, 10 mM HEPES, and 5 mM EGTA (pH 7.3 using KOH, 305 mOsm). The bath solution contained 142 mM NaCl, 8 mM KCl, 1 mM CaCl_2_, 6 mM MgCl_2_, 10 mM glucose, and 10 mM HEPES (pH 7.4 using NaOH, 325 mOsm). Recordings were low-pass filtered at 2.9 kHz and digitized at 10 kHz with an EPC-10 amplifier. Analyses were performed using PatchMaster and FitMaster software (HEKA Elektronik, Harvard Bioscience, MA, USA). Voltage-gated currents were induced via depolarizing steps in increments of 10 mV from a holding potential of −70 mV to +40 mV. Miniature postsynaptic currents (mPSCs) of MSNs, suggesting mainly GABAergic inhibitory synaptic activity [11,12], were recorded at a holding potential of −70 mV in voltage-clamp mode. For quantification only, mPSCs peaks between 10–150 pA were analyzed to exclude noise artifacts (<10 pA) and action-potential-related currents (>150 pA). Elicited and spontaneous action potentials (APs) were monitored in the current-clamp mode at holding potentials of −50 mV to −70 mV. Visual selection of medium-sized multipolar neurons had yielded >90% of DARPP-32 positive MSNs in a previous study [12], thus, the MSNs were similarly identified by their typical morphology for the electrophysiological analyses. Functional properties were recorded from three controls and MSA-P lines after at least three independent differentiations.

### 2.11. Statistical Analyses

GraphPad Prism 5 Software (GraphPad Software, San Diego, CA, USA) was used to perform the statistical analyses. Data from MSN lines were pooled in two groups (MSA-P and healthy control) and are presented as mean ± standard error of the mean (SEM). Nonparametric Mann–Whitney-U test was applied for Data with no normal distribution. When normal distribution was given, a two-tailed unpaired *t*-test test was calculated comparing the control group with the MSA-P group. When more than one group was compared to one control group, a one-way ANOVA followed by Dunn’s multiple comparisons post hoc test was applied, and for multiple comparisons, two-way ANOVA followed by Bonferroni post hoc analysis was used. The significance level (*p*-value) was set to *p* < 0.05 with * *p* < 0.05, ** *p* < 0.01, *** *p* < 0.001.

## 3. Results

### 3.1. Generation of iPSC Lines from MSA-P Patients and Healthy Controls

**Figure 1:**
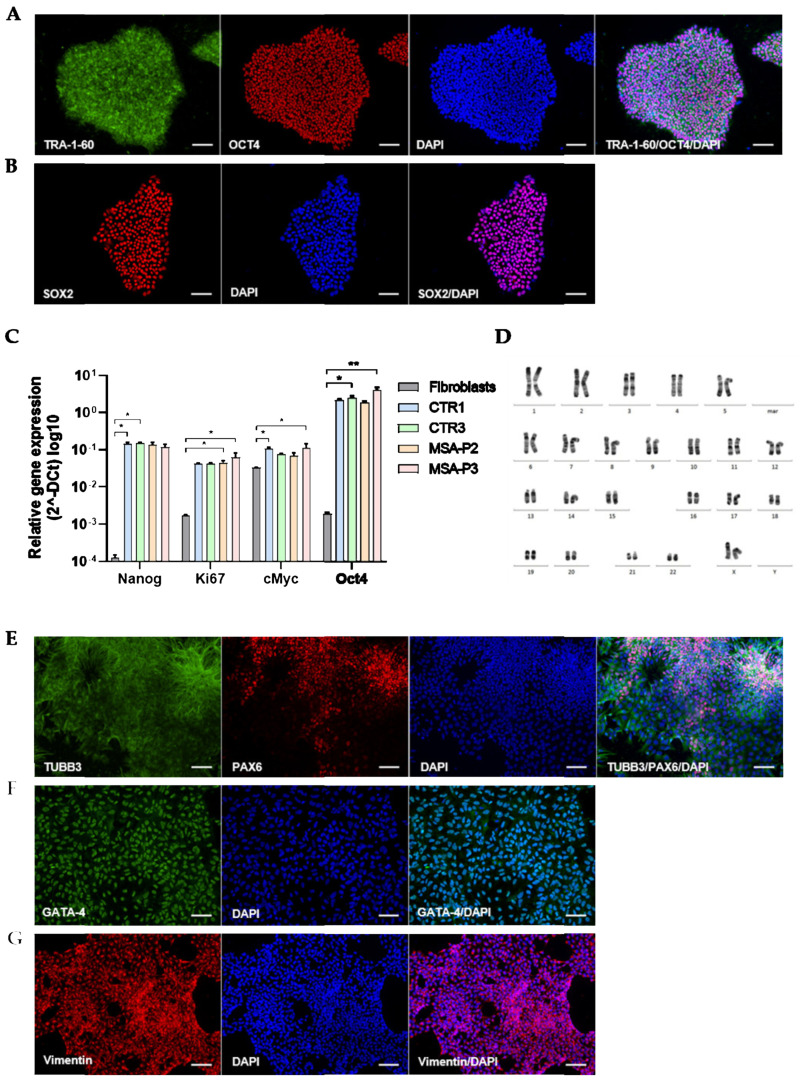
Characterization of iPSC lines. All MSA-patient (*n* = 2) and healthy control (*n* = 2) lines (newly generated in this study) expressed pluripotency markers podocalyxin (TRA-1-60), octamer-binding transcription factor 4 (OCT4, cell line MSA-P2) (**A**), and the sex-determining region Y-box 2 (SOX2, cell line MSA-P3) (**B**), as detected by immunochemical stainings. (**C**) qRT-PCR revealed increased expression of pluripotency markers in all novel iPSC lines when compared to their cell type of origin—human adult skin fibroblasts (* *p* < 0.05, ** *p* < 0.01, Data were subjected to Kruskal–Wallis testing, followed by Dunn‘s multiple comparisons post hoc test). (**D**) All generated iPSC lines exhibited euploidy, as confirmed by single nucleotide polymorphism array-based karyotyping, as represented by the MSA-P3 line. (**E**–**G**) To confirm pluripotency, iPSC lines were differentiated in vitro into cells expressing markers of all three germ layers: (**E**) TUBB3 and PAX6 for ectoderm (cell line CTR1), (**F**) GATA-4 for endoderm (cell line MSA-P2), and (**G**) Vimentin for mesoderm (cell line MSA-P2). Nuclei were counterstained with 4,6-diamidino-2-phenylindole (DAPI). Scale bars indicate 75 µm.

Additional iPSC lines from two MSA-P patients (probable diagnosis using Gilman criteria [1]) and two age- and sex-matched healthy controls (Table 1) were generated for the molecular and functional analyses. The successful reprogramming was confirmed using multiple assays. All four generated iPSC lines expressed the pluripotency markers octamer-binding transcription factor (OCT) 4, sex-determining region Y-box (SOX) 2, and podocalyxin (TRA-1-60), as shown by the immunochemical stainings (Figure 1A,B). The quantitative real-time PCR (qRT-PCR) results demonstrated significantly elevated gene expression of the pluripotency markers when compared to their origin fibroblast cell type (Figure 1C). The directed differentiation into each of the three germ layers (mesoderm, endoderm, and ectoderm) resulted in the cells expressing vimentin and GATA binding protein 4 (GATA-4), as well as paired box protein Pax-6 (PAX6) and β-tubulin III (TUBB3), respectively (Figure 1E–G). The euploidy of the generated iPSC lines was confirmed using single nucleotide polymorphism array-based karyotyping. A representative image of the karyotype of the iPSC line MSA-P3 is shown in Figure 1D.

### 3.2. Differentiation of iPSCs into Striatal Medium Spiny Neurons (MSNs)

**Figure 2:**
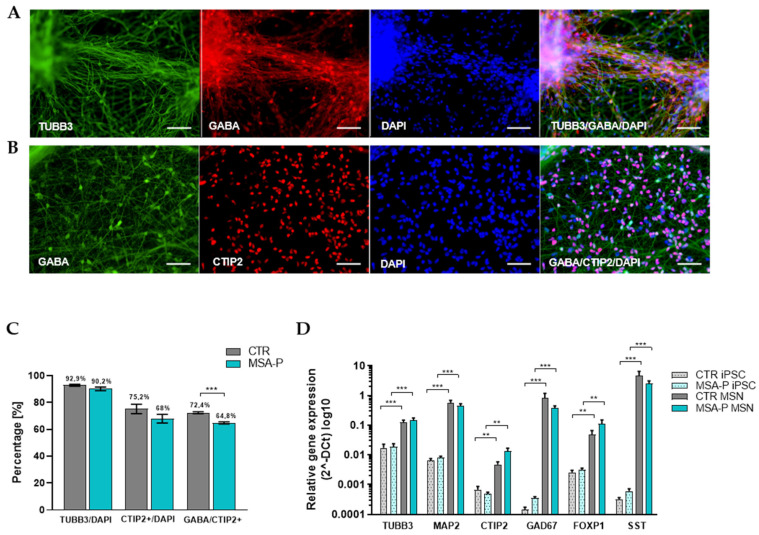
Differentiation of iPSC-derived striatal medium spiny neurons (MSNs) from MSA-P patients and healthy controls. (**A**,**B**) Immunocytochemical stainings of mature MSNs at day 70 (±3 days) of differentiation, confirming the expression of the neuronal markers β-tubulin III (TUBB3), γ-aminobutyric acid (GABA), and MSN-specific striatal marker COUP TF1-interacting protein 2 (CTIP2, cell line MSA-P3). Nuclei were counterstained with DAPI. Scale bars indicate 50 µm. (**C**) ~90% of cells in the cultures expressed the neuronal marker TUBB3. A total of 75% of the DAPI-stained control MSNs and 68% of the MSA-P MSNs expressed CTIP2 (nonparametric Mann–Whitney test. *p* = 0.07). A total of 72% (CTR) and 65% (MSA-P) of the CTIP2-positive cells coexpressed GABA (nonparametric Mann–Whitney test. *** *p* < 0.001). (**D**) Expression analysis of the iPSCs and MSNs derived from the MSA-P patients and healthy controls by quantitative real-time PCR at day 70 (±3 days) of differentiation. The MSA-P and control MSNs expressed significantly elevated levels of neuronal markers (FOXP1, forkhead box protein P1; TUBB3, b-tubulin III; MAP2, microtubule-associated protein 2), a GABAergic marker (GAD67, glutamic acid decarboxylase), a striatal interneuron marker somatostatin (SST), and the MSN-specific striatal marker (CTIP2, COUP TF1-interacting protein 2) when compared to the iPSCs. The quantitative expression of neuronal, GABAergic, interneuron, and MSN-specific markers was similar for MSA-P and the control MSNs. Data are presented as means ± SEM in a logarithmic scale (log10) from at least three independent differentiations for the control and MSA-P lines (** *p* < 0.01, *** *p* < 0.001, parametric *t*-test or nonparametric Mann–Whitney test).

Since neuronal pathology is very prominent in the putamen in MSA, we differentiated the iPSCs from three MSA-P patients and three matched healthy controls for 70 days into MSNs, which is the most frequent neuronal cell type in the striatum. Following our differentiation protocol (published previously [11]), we generated around 90% β-III tubulin (TUBB3) positive and 68–75% COUP TF1-interacting protein 2 (CTIP2)-positive neurons, of which 91–93% coexpressed the neurotransmitter γ-aminobutyric acid (GABA) (Figure 2A–C, Supplementary Figure S1). Significantly more CTIP2-positive CTR MSNs were stained positive for GABA (*p* < 0.001), resulting in a larger population of CTIP2/GABA coexpressing MSNs when compared to the cultures of the MSA-P patients. There was a significant upregulation of mRNA expression of the neuronal and striatal markers when comparing the MSNs to the iPSC origin (shown by qRT-PCR), but this was not significantly different between the healthy controls and the MSA-P MSNs (Figure 2D). When emphasizing the immunostaining results, the significant upregulation of the mature neuronal marker microtubule-associated protein 2 (MAP2), and with MSN maturation associated markers glutamic acid decarboxylase (GAD67) and transcription factor forkhead box protein P1 (FOXP1) [25], the neuronal and mainly GABAergic phenotype of the differentiated MSNs was confirmed. Additionally, the significantly elevated genomic expression of somatostatin (SST) in the MSNs compared to the iPSCs indicates the presence of striatal GABAergic interneurons in our cell culture (Figure 2D).

### 3.3. Functional Properties of MSNs

#### 3.3.1. Calcium Signaling in Differentiated MSNs

**Figure 3:**
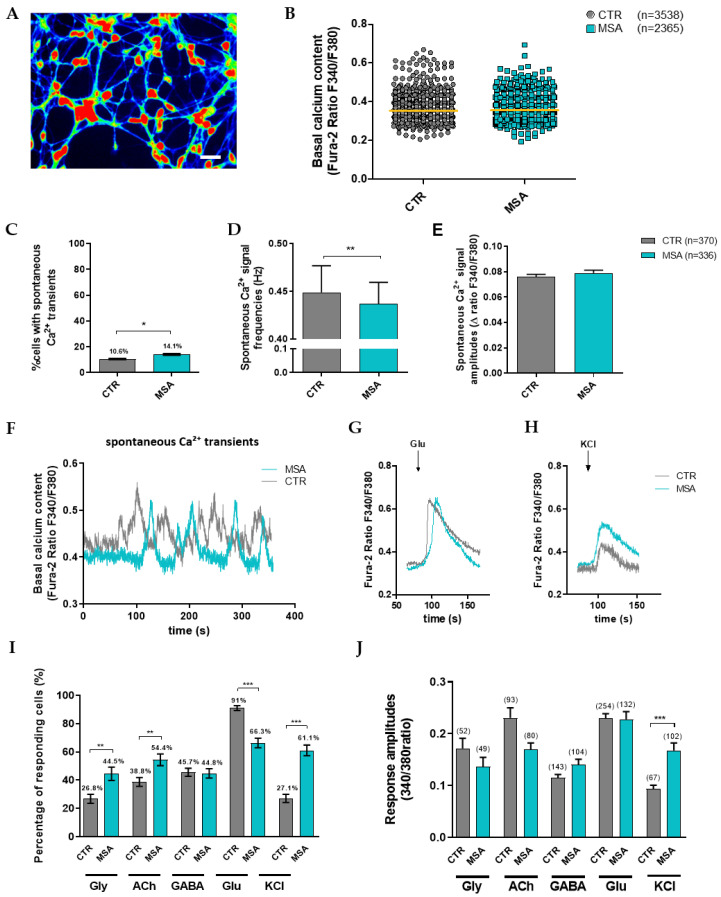
Spontaneous and transmitter-induced calcium (Ca^2+^) signaling of MSNs derived from patients with MSA-P and healthy controls at day 70 (±4 days) of differentiation. Intracellular Ca^2+^ transients are presented as ratios of the fluorescence signals obtained at 340 and 380 nm (F_340_/F_380_). (**A**) Representative image of Ca^2+^ recording of Fura-2-loaded MSA-P MSNs. Scale bar indicates 25 µm; (**B**) Basal intracellular Ca^2+^ did not differ between the MSA-P MSNs (*n* = 2365) and the control MSNs (*n* = 3538). (**C**) A significantly higher percentage of MSA-P MSNs (14.1%, *n* = 2369), rather than control MSNs (10.6%, *n* = 3477), exhibited spontaneous Ca^2+^ transients (*p* = 0.0251, nonparametric Mann–Whitney test). (**D**) The frequency of the spontaneous Ca^2+^ transients was significantly lower in the MSNs derived from the MSA-P patients (0.437 Hz, *n* = 335) than in those from the healthy controls (0.449 Hz, *n* = 372, *p* = 0.0047, nonparametric Mann–Whitney test). (**E**) Spontaneous Ca^2+^ amplitudes of the MSNs were not significantly different between the groups (MSA-P *n* = 336, CTR *n* = 370). (**F**) Representative traces of the spontaneous Ca^2+^ peaks of the control and MSA-P MSNs. Representative traces of intracellular Ca^2+^ changes of the Fura-2-loaded MSA-P and control neurons induced by the separate application of (**G**) glutamate (Glu, 50 µM) and (**H**) KCl (50 mM). (**I**) The percentage of neurons with the Ca^2+^ transients after separate application of the transmitter glycine (Gly, 100 µM), acetylcholine (ACh, 100 µM), and KCl (50 mM) was significantly elevated in the MSNs from the MSA-P patients, compared to the healthy controls (Gly: MSA-P 44.5%, CTR 26.8%, *p* = 0.0022; ACh: MSA-P 54.4%, CTR 38.8%, *p* = 0.0045; KCl: MSA-P 61.1%, CTR 27.1%, *p* < 0.0001), whereas the percentage of responding MSA-P MSN was significantly (*p* < 0.0001) reduced after the administration of glutamate (Glu, 50 µM, MSA-P 66.3%, CTR 91%). The percentage of cells responding to GABA (100 µM) did not differ between the groups (~45%). (**J**) Cytosolic Ca^2+^ response amplitudes were similar for both groups upon the separate application of the neurotransmitters Gly, ACh, GABA, and Glu. Only the application of KCl led to a significantly higher response amplitude in MSA-P MSN than in the control MSNs (*p* < 0.0001, nonparametric Mann–Whitney test). Data from three control and MSA-P lines from at least three independent differentiations were analyzed. * *p* < 0.05, ** *p* < 0.01, *** *p* < 0.001. Data are presented as means ± SEM.

Principally, the cells with multipolar dendritic morphology (Figure 3A) were chosen to assess their basal calcium content and spontaneous Ca^2+^ transients, as well as their responsiveness to various neurotransmitters. Two to four independent differentiations (70 ± 4 days) per cell line were assessed. The basal content of the Fura-2-loaded MSNs did not differ between the MSA-P patients and the healthy controls (Figure 3B, Supplementary Figure S2a), but a significantly (*p* = 0.0251) larger percentage of the MSA-P MSNs (14.1%) elicited the spontaneous Ca^2+^ transients when compared to the control cells (10.6%, Figure 3C, Supplementary Figure S2b). In contrast, the frequency (Figure 3D,F, Supplementary Figure S2c) of the Ca^2+^ transients was significantly (*p* = 0.0047) reduced in the MSNs from the MSA-P patients, whereas the amplitude (Figure 3E,F, Supplementary Figure S2d) did not show significant differences compared to the control MSNs.

#### 3.3.2. Altered Responsiveness to Neurotransmitters and KCl in MSA-P MSNs

The percentage of responding cells was significantly elevated in the MSA-P MSNs after the application of glycine (CTR 26.8% vs. MSA-P 44.5%, *p* = 0.0022), acetylcholine (CTR 38.8% vs. MSA-P 54.4%, *p* = 0.0045), and potassium chloride (CTR 27.1% vs. MSA-P 61.1%, *p* < 0.0001), whereas there was no difference after the application of GABA between the groups (CTR 45.7% vs. MSA-P 44.8%, Figure 3I). In contrast, the percentage of responding cells was significantly (*p* < 0.0001) reduced in MSA-P MSN (66.3%) compared to the healthy controls (91%) after applying glutamate (Figure 3I). The amplitude of the Ca^2+^ transients in response to the application of KCl was significantly (*p* < 0.0001) elevated in MSA-P (0.17 ± 0.015) compared to the control MSNs (0.09 ± 0.007, Figure 3H,J), whereas the Ca^2+^ peaks elicited by the application of any other neurotransmitter were similar in both groups (glycine: MSA-P 0.14 ± 0.018, CTR 0.17 ± 0.019; acetylcholine: MSA-P 0.17 ± 0.012, CTR 0.23 ± 0.02; GABA: MSA-P 0.14 ± 0.01, CTR 0.12 ± 0.006; glutamate: MSA-P 0.23 ± 0.015, CTR 0.23 ± 0.009, Figure 3G,J).

#### 3.3.3. Characteristics of Voltage-Gated ion Channels and Evoked Action Potentials

**Figure 4:**
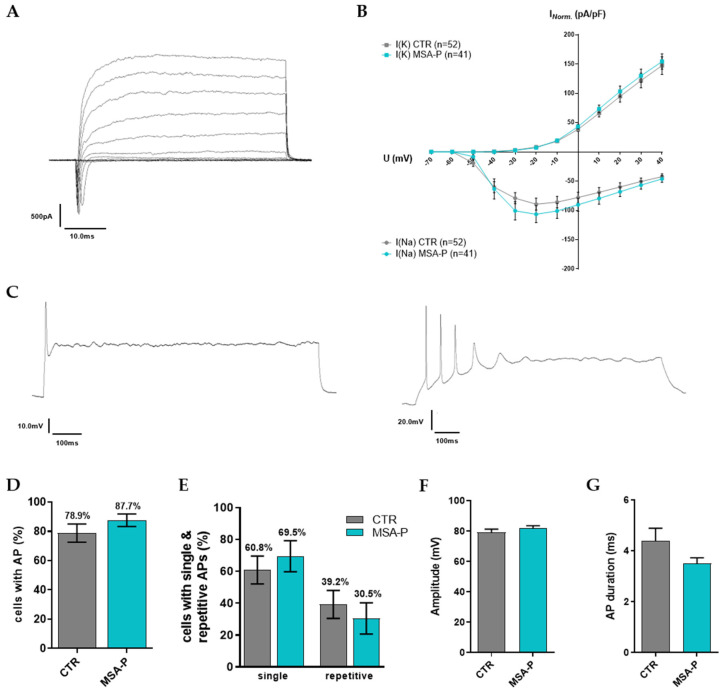
Voltage-gated ion currents, as well as elicited action potentials (APs), of the MSNs derived from the MSA-P patients and healthy controls at day 70 (±3 days) of differentiation. (**A**) Sodium inward and potassium outward currents of MSA-P MSN after maturation for 70 days were recorded in whole-cell voltage-clamp mode via depolarizing steps in increments of 10 mV from a holding potential of −70 mV to +40 mV. (**B**) IV-plot indicating that the ion current amplitudes (normalized for individual cell sizes based on the capacitances of the cell membrane (pA/pF)) were not significantly different between MSA-P MSNs (*n* = 41) and the controls (*n* = 52, two-way ANOVA with Bonferroni post hoc test). (**C**) Examples of MSA-P MSNs spiking single and repetitive APs upon depolarization from holding potentials of −60 mV to −70 mV. (**D**) The percentages of the neurons firing APs during depolarizations were not significantly different between the MSA-P MSNs and the controls (nonparametric Mann–Whitney test). (**E**) The percentages of neurons with single or repetitive (2, 3, 4, ≥ 5) APs were comparable between the MSNs of both groups (nonparametric Mann–Whitney test). (**F**) AP peak amplitude and (**G**) duration were not significantly different between MSA-P (*n* = 35) and the control MSNs (*n* = 44, unpaired *t*-test or nonparametric Mann–Whitney test). Data were analyzed from three healthy control and MSA-P cell lines after at least three independent differentiations and are presented as means ± SEM.

At least three independent differentiations of the mature MSNs (70 days ± 3 days) from three MSA-P patients and three healthy control iPSC lines were measured using whole-cell patch-clamp recordings to assess synaptic functionality, the passive membrane properties, action potentials, and the voltage-gated ion channels (Table 2), evaluating neuronal excitability. The MSNs from both the MSA (*n* = 41) and the control group (*n* = 52) exhibited large sodium inward and potassium outward currents induced by depolarizing 10 mV voltage increments from a holding potential of −70 mV to +40 mV (Figure 4A). The peak current amplitudes were normalized according to cell size based on the cell membrane capacitance (pA/pF, Figure 4B), showing no significant difference in the I/U-plot. Additionally, both groups did not significantly differ in resting membrane potential, membrane capacitance, and input resistance (Table 2, Supplementary Figure S3).

Representative traces of the evoked single and repetitive action potential (AP) firing are shown in Figure 4C. A total of ~79% of the MSNs were differentiated from the healthy control iPSC lines, and ~88% of the MSA-P MSNs fired single APs during the depolarizing current injections (Table 2, Figure 4D, Supplementary Figure S4a). No significant difference was found for evoked repetitive spiking, which was less frequently recorded in MSA-P (30.5%) than in the control MSNs (39.2%) (Figure 4E, Supplementary Figure S4b,c). The amplitudes (MSA-P 81.8 ± 1.8 mV, CTR 79.2 ± 2.2 mV, Figure 4F, Supplementary Figure S4d) and the duration of the evoked APs (MSA-P 3.5 ± 0.2 ms, CTR 4.4 ± 0.5 ms, Table 2, Figure 4G, Supplementary Figure S4e) were comparable in both groups. The amplitude of afterhyperpolarization (AHP, MSA-P 12.1 ± 0.84 mV, CTR 11.7 ± 1 mV), as well as the time to peak AHP (MSA-P 17.5 ± 1.8 ms, CTR 18.2 ± 1.7 ms), were similar (Table 2).

#### 3.3.4. MSA-P MSNs Exhibit Reduced Spontaneous AP Frequency and Synaptic Activity

**Figure 5:**
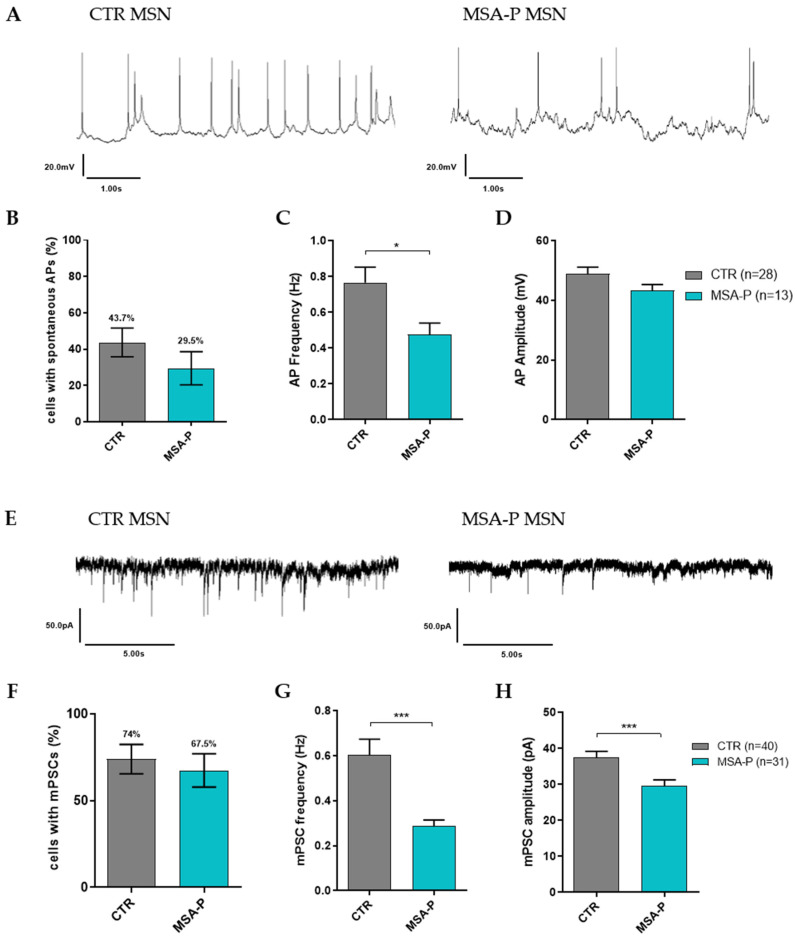
Spontaneous action potentials (APs) and synaptic activity of MSNs from MSA-P patients and healthy controls at day 70 (±3 days) of differentiation. (**A**) Representative traces of spontaneous APs from both groups. (**B**) Percentage of neurons with spontaneous APs was similar for MSA-P and control MSNs. (**C**) The frequency of spontaneous APs was significantly reduced in the MSNs from the MSA patients compared with the MSNs from healthy controls (*p* = 0.0291, nonparametric Mann–Whitney test), whereas the amplitudes did not differ between the groups (**D**). (**E**) Representative traces of the mPSCs from MSA-P and the control MSNs. (**F**) Similar to the spontaneous APs, the percentage of neurons showing miniature postsynaptic currents (mPSCs) as synaptic activity was not significantly different between MSA-P and the control MSNs. (**G**) However, the frequency, as well as (**H**) the amplitude, of the mPSCs were significantly reduced in the MSA-P MSNs when compared to the control MSNs (*p* = 0.0007 and *p* < 0.0001, nonparametric Mann–Whitney test). Data from three control and MSA-P lines from at least three independent differentiations were analyzed. * *p* < 0.05, *** *p* < 0.001. Data are presented as means ± SEM.

The percentage of cells with spontaneous AP firing was lower in MSA-P (29.5%) than in the control MSNs (43.7%) (Figure 5B, Supplementary Figure S5a). The frequency was significantly (*p* = 0.0291) reduced in those MSNs differentiated from the MSA-P patients (0.48 ± 0.06 Hz) when compared to the control lines (0.76 ± 0.09 Hz, Table 2, Figure 5C, Supplementary Figure S5b), whereas the amplitude of the spontaneous APs was comparable in both groups (MSA-P 43.3 ± 2 mV, CTR 48.8 ± 2.3 mV, Figure 5D, Supplementary Figure S5c). Representative traces of spontaneous AP firing are illustrated in Figure 5A. A similar percentage of MSNs displayed miniature postsynaptic currents (mPSCs, MSA-P 67.5%, CTR 74%), indicating the formation of functional synaptic connections (Figure 5F, Supplementary Figure S6a). However, when comparing the frequency of the mPSCs, the MSA-P MSNs (0.29 ± 0.03 Hz) showed a significant (*p* < 0.0001) reduction compared to the control MSNs (0.6 ± 0.07 Hz, Figure 5G, Supplementary Figure S6b). A similar observation could be seen regarding the amplitude of the mPSCs, with a significantly (*p* = 0.0007) lower amplitude in those MSNs differentiated from the MSA-P patients (29.5 ± 1.7 pA) than from the healthy controls (37.4 ± 1.8 pA, Table 2, Figure 5H, Supplementary Figure S6c). Figure 5E displays the representative traces of the mPSCs for MSA-P and the control MSNs.

### 3.4. Expression of GABA_A_ and GABA_B_ Receptor Subunits, ATP-Dependent K^+^ Channels (Sulfonylurea Receptors), and Voltage-Gated Ca^2+^ Channels

#### 3.4.1. Altered Expression of GABA_A_ Receptor Subunits

**Figure 6:**
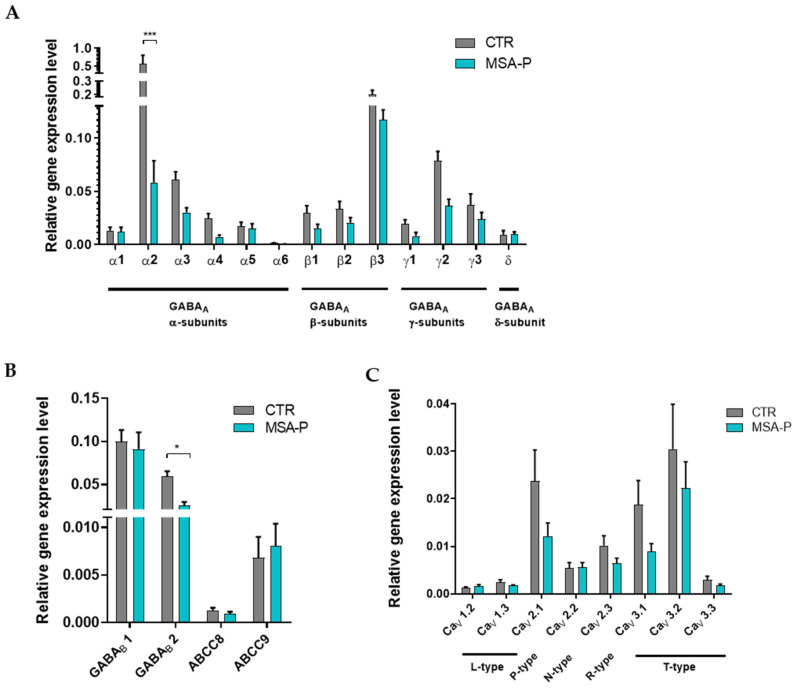
Expression analysis of ion channels by quantitative real-time PCR in the MSNs from the MSA-P patients and the healthy controls at day 70 (±3 days) of differentiation. (**A**) The genomic expression of γ-aminobutyric acid type A (GABA_A_) receptor subunits α(1–6), β(1–3), γ(1–3), and δ. For α2, the expression was significantly reduced in MSA-P neurons compared with the healthy control MSNs (*** *p* < 0.001, two-way ANOVA followed by Bonferroni post hoc analysis). (**B**) The genomic expression of γ-aminobutyric acid type B (GABA_B_) receptor subunits 1 and 2, as well as sulfonylurea receptor 1 (ABCC8) and 2 (ABCC9). MSA-P MSNs showed a significant reduction in GABA_B_2 expression compared to the control MSNs (* *p* < 0.05, two-way ANOVA followed by Bonferroni post hoc analysis). (**C**) The genomic expression of voltage-gated Ca^2+^-channels. Data from three control and MSA-P lines from at least three independent differentiations were analyzed. Data are presented as means ± SEM.

The MSA-P MSNs tended to have a reduced expression of the genes encoding the GABA_A_ receptor subunits α, β, and γ compared with the MSNs from the healthy controls, whereas the expression of the δ-subunit did not differ between the groups (Figure 6A). The only significant (*p* < 0.0001) reduction was detected for the gene encoding the α2-subunit.

#### 3.4.2. Reduced Expression of GABA_B_ Receptor Subunit 2

It was postulated that inhibited GABA transmission via the activation of ATP-dependent K^+^ channels, specifically the sulfonylurea receptor (SUR) I-K_ATP_ channels, regulates α-synuclein secretion in mice striatum [26]. Therefore, we investigated the expression of genes for the sulfonylurea receptors I and II (SUR1 and SUR2, encoded by ABCC8 and ABCC9, respectively), as well as GABA_B_ receptor subunits 1 and 2 that are hypothesized to also be expressed in nerve terminals releasing α-synuclein [26]. The expression of the GABA_B_ receptor subunit 2 was found to be significantly (*p* < 0.05) reduced in the MSA-P MSNs at day 70 ± 3 days, compared with the control MSNs, whereas the expression of the GABA_B_ receptor subunit 1, as well as SUR1 and SUR2, did not differ between the groups (Figure 6B).

#### 3.4.3. Comparable Expression of Voltage-Gated Ca^2+^ Channel Subtypes

The genomic expression levels of the voltage-gated Ca^2+^ channel subtypes, which are downstream and are also involved in the GABA_B_ receptor-associated release of α-synuclein [26], tended to be reduced but did not significantly differ in MSA-P when compared to the control MSNs (Figure 6C), suggesting that these channels do not contribute markedly to the α-synuclein pathology in MSA-P.

### 3.5. α-Synuclein Characteristics in MSNs

#### 3.5.1. Elevated Release of α-Synuclein by Differentiated MSA-P MSNs

**Figure 7:**
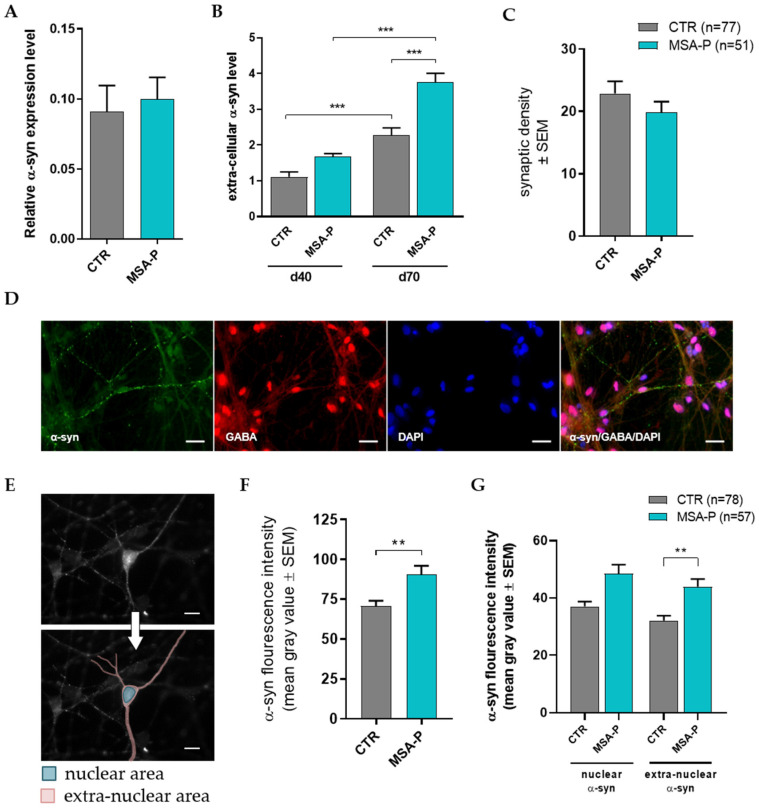
α-Synuclein (α-syn) characteristics in the MSNs of three MSA-P patients and three healthy controls. (**A**) Relative gene expression of α-synuclein in mature MSNs was similar for MSA-P and the control cell lines at day 70 (±3 days) of differentiation. (**B**) The release of α-synuclein into the medium, as observed using dot blot analysis, was significantly (*p* < 0.001) elevated in the mature (d70) MSA-P MSNs when compared to the immature (d40) MSA-P MSNs and the mature control MSNs. Additionally, the mature control MSNs revealed higher α-synuclein release than the immature control MSNs. Data are presented as means ± SEM. Data were subjected to one-way ANOVA, followed by Bonferroni’s multiple comparisons post hoc test. At day 40 (±4 days) and at day 70 (± 4 days), conditioned medium was used. (**C**) No significant difference in the number of GABAergic synapses in MSA-P and the control MSNs at day 70 (±3 days) of differentiation was observed in morphometric analysis. (**D**) The distribution of α-synuclein within the MSNs was quantified via immunocytochemistry. Representative images of the mature MSNs of the cell line MSA-P3 at day 70 (±3 days) of differentiation, showing the distribution of α-synuclein in GABA-positive neurons. Scale bars indicate 25 µm. (**E**) Representative image of α-synuclein staining and tracing of what is considered the nuclear area (blue) and the extranuclear area (red). Scale bars indicate 20 µm. (**F**) Quantitative morphometric analysis revealed a significantly higher amount of α-synuclein in the MSA-P MSNs compared to the control MSNs (*p* = 0.0029, nonparametric Mann–Whitney test). (**G**) The supply of extranuclear α-synuclein was significantly (*p* = 0.002) elevated in the MSA-P MSNs, whereas the difference in nuclear α-synuclein did not quite reach a significant difference. Data are presented as means ± SEM. Data were subjected to Kruskal–Wallis testing, followed by Dunn‘s multiple comparisons post hoc test. ** *p* < 0.01, *** *p* < 0.001.

The mRNA expression of α-synuclein did not differ between MSA-P and the healthy control MSNs (Figure 7A), as was shown by quantitative RT-PCR. The capability of the MSNs to release α-synuclein into the extracellular space was evaluated by dot blot analysis. It revealed a significant (*p* < 0.0001) increase in α-synuclein release from the MSA-P MSNs at day 70 compared to the control MSNs at the same stage of differentiation (Figure 7B). As expected, the release was significantly lower in the less mature MSNs for both groups, suggesting a not yet fully mature functionality of MSNs at day 40.

#### 3.5.2. Unaltered GABAergic Synaptic Density

In order to rule out the possibility of the altered synaptic density in the MSA-P MSNs being responsible for the reduced synaptic activity or increased α-synuclein release, a morphometric evaluation of synapse formation was performed, revealing comparable synaptic densities for the GABA-positive boutons in the MSA-P MSNs and controls (Figure 7C).

#### 3.5.3. MSA-P MSNs Contain Significantly More α-Synuclein Than Control MSNs

Assessing the amount and distribution of α-synuclein in the MSNs using GABA and α-synuclein double immunostaining (Figure 7D) revealed a significantly (*p* = 0.0029) elevated amount of α-synuclein in the neurons derived from the MSA-P patients compared to the controls (Figure 7F). The extranuclear burden of α-synuclein was significantly (*p* = 0.002) higher in the MSA-P neurons, whereas the difference in the nuclear area did not reach significant levels when compared to the MSNs derived from the healthy controls (Figure 7G).

## 4. Discussion

The understanding of the cellular and molecular pathology of MSA is still limited. Difficulties result from the rare availability of postmortem brain tissue from MSA patients and the establishment of suitable animal or human cell models for the disease. Recent studies using O4^+^ oligodendrocyte lineage cells [27] and dopaminergic neurons [14,28] generated from MSA patient-derived iPSCs set a promising foundation. Since MSA-P is also associated with the marked degeneration of GABAergic striatal MSNs [10], we adapted our previously established protocols for the differentiation of the iPSCs toward striatal neurons [11,12] to study the pathophysiology of the disease, which had not been carried out before in iPSC-derived striatal MSNs. Therefore, the skin biopsies of the MSA-P patients and the matched, healthy controls were taken to establish a patient-specific MSN model.

After 70 days, the majority of the differentiated neurons expressed various neuronal markers, as well as the striatal marker CTIP2, as shown by quantitative RT-PCR and immunocytochemistry. The MSA-P neurons showed a significantly reduced percentage of GABA/CTIP2 double-positive cells, suggesting a reduced differentiation efficiency in the striatal MSNs in the patient cell lines. As reported previously [16], we not only found CTIP2-positive MSNs but also a population of somatostatin-positive neurons, indicating the additional presence of striatal GABAergic interneurons in our cultures.

Calcium signaling plays a fundamental role in various aspects of neuronal function, and dysregulated calcium homeostasis was found to play a role in the pathogenesis of PD [29,30], Huntington’s disease [31], Alzheimer’s disease [32,33,34], and different types of dystonias [12,16,22]. In order to identify any possible involvement of altered intracellular calcium dynamics in the pathology of multiple systems atrophy, we performed Ca^2+^ imaging in the differentiated striatal neurons of the MSA-P patients and the healthy control subjects. Some studies of neurodegenerative diseases and movement disorders describe an increase in basal calcium levels in the affected neuronal cells [16,30,35], whereas we could not observe this in the striatal neurons of the MSA-P patients. These elevated levels of intracellular calcium are often linked to increased ER stress [30,36,37]. When examining the mRNA expression of the UPR signaling markers (CHOP, ATF4, GRP94, and HSPA5), we were unable to detect any differences between the MSA-P patients and the healthy controls (data not shown), facilitating the assumption that this common mechanism in neurodegeneration does not play a major part yet in the manifestation of MSA pathology in striatal neurons at this stage of maturation and disease progression. Besides, we found a significant reduction in the frequency of spontaneous calcium transients in the MSA-P MSNs, although the expression of voltage-gate calcium channels was similar when compared to the healthy controls. This suggests that Ca^2+^ homeostasis is disturbed in MSA-P MSNs, which might add to their functional pathophysiology.

In consonance with the reduced frequency of spontaneous Ca^2+^ transients in MSA-P MSNs, the frequency of spontaneous action potentials and synaptic activity (miniature PSCs) was also significantly reduced in these neurons, as seen in the whole-cell patch-clamp recordings, indicating a pathological hypoexcitability phenotype. The functional outcome with decreased activity for the MSA-P MSNs might eventually result in the reduced GABAergic inhibition of striatal MSN projection targets. In a previous study with α-synuclein overexpression in mice, a significant reduction in spontaneous synaptic activity in MSNs, as early as 35 days of age and persisting up to an age of 300 days, was found with the independency of this reduction in spontaneous EPSC frequencies from presynaptic action potentials [38]. Similar observations were made in striatal MSNs from an HD mouse model [39]. In these mice, progressive reductions in the frequency of spontaneous EPSCs began at 5–7 weeks and became more pronounced by 11–15 weeks of age, in correlation with the progression of behavioral symptoms. Both studies suggested a correlation between this activity deficit and reduced presynaptic glutamate release in the mouse striatum. Using calcium imaging, we found alterations in the percentage of responding cells upon transmitter application with a significant reduction in the responding cells of the MSA-P patients upon glutamate application, whereas the amplitude of the Ca^2+^ transients induced by glutamate did not differ between the patient and control MSNs. Some MSA-P and control MSNs had a small GABA-evoked calcium transient, indicating neuronal depolarization and the indirect activation of voltage-gated calcium channels. Due to the switch of GABA_A_ receptor-mediated actions from de- to hyperpolarization in early brain maturation [40,41,42,43,44,45], we expect to see the presence of these differential GABA effects during MSN differentiation too. In human iPSC-derived neuronal organoids, this developmental GABA polarity switch was reported after 40 days, indicating enhanced network maturation [46].

In the striatal MSA-P MSNs, we found a significant reduction in the expression of the GABA_A_ receptor subunit α2, as was also observed recently in the MSNs of THAP1 dystonia patients [16]. Therefore, a reduction in this GABA_A_ receptor subunit might add to the significantly lower frequency of the mPSCs in the MSA patient cells that showed a similar synaptic density to the controls. In contrast to the disinhibited THAP1 MSNs, the hypoexitability of MSA-P MSNs suggests a more profound functional pathophenotype, which predominates despite the marked decrease in the GABA_A_ receptor subunit α2. Additionally, we also found a significant reduction in the expression of the GABA_B_ receptor subunit 2, which might also contribute to the pathophysiology of GABAergic transmission in MSA neurons.

In our striatal neurons, the genomic expression of α-synuclein was not significantly higher in the MSNs of the MSA-P patients than in the healthy controls. A previous study [14] also showed a similar amount of α-synuclein protein in the iPSC-derived dopaminergic neurons from MSA patients and controls. A mild but not significant decrease in the mRNA expression of α-synuclein was detected in neurons isolated from MSA brains [47]. Interestingly, they also observed slightly increased expression of the SNCA gene within oligodendrocyte lineage cells [47].

Although we did not find a difference in SNCA mRNA expression, a significantly higher amount of α-synuclein was detected in the MSNs derived from the MSA-P patients in the immunostainings. This increased α-synuclein burden was more pronounced in the extranuclear area of the neurons than in the nuclear area. Even though the same amount of SNCA mRNA is expressed, the MSA-P MSNs seem to translate more α-synuclein, which then is distributed to the neurites of the cells from where it might be released.

It was demonstrated previously that oligodendroglial cell lines take up α-synuclein monomers, oligomers, and, to a lesser extent, also fibrils [48,49], giving a first hint for in vivo neuron-to-oligodendrocyte transfer of α-synuclein [48]. This assumption is supported by our finding that, in the medium of mature MSA-P MSNs, a significantly higher amount of α-synuclein was detected than in the medium of the MSNs derived from healthy controls. This leads to the hypothesis that MSNs from MSA-P patients release pathological amounts of α-synuclein into the extracellular space, where it is thereafter taken up by neighboring oligodendrocytes, resulting in the formation of GCIs. Interestingly, the premature MSNs did not release the same amount of α-synuclein, suggesting that the mechanisms leading to this pathological release are not yet fully developed during MSN differentiation.

The α-synuclein secretion in the mouse striatum is thought to be regulated by GABA transmission via SUR1-K_ATP_ channels and GABA_B_ receptors [26]. Activation of the SUR1-K_ATP_ channels located on the membrane of the GABAergic neurons causes membrane hyperpolarization and a decrease in GABA release. Locally, lower GABA levels reduce the activation of the GABA_B_ receptors on neighboring glutamatergic nerve endings, compromising the inhibition of the associated Ca^2+^ channels. The resulting rise in intracellular Ca^2+^ then triggers α-synuclein release [26].

Therefore, we examined the expression of GABA_B_ receptors and observed a significantly reduced expression of the subunit 2 in the MSA-P neurons compared to the healthy controls. The downregulation of this GABA_B_ receptor subunit 2 in our MSA-P neurons potentially reinforces the GABA mediated pathomechanisms of α-synuclein release, contributing to the hypoexcitability measured in the electro-physiological recordings.

GABA_B_ receptors are also known to be expressed on the axon terminals of striatal SST^+^ interneurons [50], which can generate excitation–inhibition sequences in MSNs due to the corelease of glutamate and GABA [51]. The binding of SST to its receptors suppresses the activity of its target cells by inhibiting Ca^2+^ influx [52]. Interestingly, GABA release inhibits the spontaneous release of SST and GABA via the GABA_B_ receptors. Since we observed a significant downregulation of GABA_B_ receptor subunit 2 mRNA in our MSA-P neurons, it can be speculated that this negative feedback inhibition is insufficient. Thereby, more SST could be released by the GABAergic interneurons, amplifying the activity suppression in MSNs. Interestingly, SST-positive inhibitory interneurons are involved in marking the confines of learned movement sequences [53]. So, a decrease in this GABAergic interneuron activity might further contribute to the development of motor symptoms in MSA-P.

In conclusion, our data of MSA-P patient-derived striatal neurons provide a feasible human in vitro model of the disease. The MSA-P MSNs showed significant hypoexcitability that was observed in both the whole-cell patch-clamp and Ca^2+^ imaging analyses. This functional pathophenotype might partly be explained by the reduced expression of GABA_A_ receptor subunit α2 and GABA_B_ receptor subunit 2, as well as by the increased burden of α-synuclein in the extranuclear area of these neuronal cells. Our study supports the hypothesis that the formation of GCIs may be a secondary process in the disease course [5] and that a pathological amount of α-synuclein could be released by striatal neurons from where it is likely to spread to neighboring cell types. Further experiments in human stem cell models of MSA-P, including the generation of oligodendrocytes and monitoring their uptake of previously released α-synuclein, might help in elucidating the still unclear disease pathomechanisms.

## Figures and Tables

**Table 1:** List of iPSC lines newly generated and used in this study.

ID Code	Diagnosis	Sex	Age at Biopsy	Previously Published
CTR1	CTRL	M	59	newly generated
CTR2	CTRL	M	59	StemBANCC consortium SFC084-03-02-01A
CTR3	CTRL	F	62	newly generated
P1	Probable MSA-P	F	78	[13]
P2	Probable MSA-P	M	52	newly generated
P3	Probable MSA-P	F	56	newly generated

**Table 2:** The electrophysiological properties recorded by the whole-cell patch-clamp of the iPSC-derived MSNs from three MSA-P lines and three healthy controls. Recordings were performed at day 70 (±3 days) of differentiation.

	Control				MSA-P			
Functional Properties	CTR1 (*n* = 23)	CTR2 (*n* = 13)	CTR3 (*n* = 19)	CTR (*n* = 55)	P1 (*n* = 14)	P2 (*n* = 13)	P3 (*n* = 14)	MSA-P (*n* = 41)
I_Na_ max. amplitudes (pA/pF)	−70.64 ± 7.1	−85.0 ± 8.9	−97.3 ± 9.9	−89.4 ± 9.1	−80.39 ± 8.8	−137.4 ± 14.7	−105.1 ± 11.2	−106.6 ± 11.3
I_K_ max. amplitudes (pA/pF)	139.9 ± 14.6	126.9 ± 13.3	152.4 ± 15.8	147.4 ± 15.3	125.6 ± 13,5	163.2 ± 17.2	174.5 ± 18.4	154.2 ± 16.4
Resting membrane potential (mV)	−29.6 ± 2.3	−23.4 ± 2.4	−36.8 ± 3.6	−30.7 ± 1.8	−25.8 ± 2.4	−27.4 ± 1.8	−26.9 ± 3.1	−26.7 ± 1.4
Membrane capacitance (pF)	20.8 ± 2.8	21 ± 3.5	18.5 ± 2.3	20 ± 1.6	14.5 ± 2.3	19.1 ± 3.6	20 ± 3.3	17.8 ± 1.8
Input resistance (MOhm)	336.6 ± 44.4	407.1 ± 46.3	486.4 ± 91.3	406.2 ± 38.9	440.3 ± 80.1	363.8 ± 49.7	311.4 ± 50.6	373.6 ± 36.4
Cells with evoked APs (%)	81.3 ± 12	68.8 ± 18.8	82.4 ± 4	78.9 ± 6.2	87.5 ± 7.2	93.3 ± 6.7	82.2 ± 9.7	87.7 ± 4.3
Cells with single evoked APs (%)	70.8 ± 12.5	46.4 ± 3.6	56.9 ± 21.6	60.8 ± 8.8	91.7 ± 8.3	62.2 ± 23.2	47.2 ± 12.1	69.5 ± 9.5
Cells with repetitively evoked APs (%)	19.4 ± 6.7	53.6 ± 3.6	21.5 ± 6.2	39.2 ± 8.8	8.33 ± 8.33	37.8 ± 11.8	39.6 ± 12	30.5 ± 9.8
Amplitude (mV) of evoked APs	77.3 ± 3.6	85.5 ± 3.8	77.8 ± 3.4	79.2 ± 2.2	78.1 ± 3.2	84.7 ± 3.1	82.7 ± 2.7	81.8 ± 1.8
Duration (ms) of evoked APs	3.92 ± 0.45	2.83 ± 0.34	5.82 ± 1.21	4.39 ± 0.51	4.31 ± 0.42	3.38 ± 0.3	2.8 ± 0.23	3.52 ± 0.22
AHP amplitude (mV)	11.8 ± 1.4	9.6 ± 1.9	12.8 ± 2	11.7 ± 1	9.1 ± 0.66	12.9 ± 1.4	14.1 ± 1.6	12.1 ± 0.84
Time to peak AHP (ms)	17.5 ± 3.1	19.4 ± 4.3	18.3 ± 2.2	18.2 ± 1.7	19.1 ± 3.6	16 ± 2.9	17.5 ± 3.1	17.5 ± 1.8
Cells with spontaneous APs (%)	48.5 ± 14.7	29.2 ± 18.2	51.9 ± 1.9	43.7 ± 7.8	18.8 ± 18.8	31.1 ± 17.4	42.2 ± 8.9	29.5 ± 9.1
Frequency of spontaneous APs (Hz)	0.77 ± 0.15	0.6 ± 0.17	0.84 ± 0.12	0.76 ± 0.09	0.33 ± 0.11	0.76 ± 0.15	0.35 ± 0.05	0.48 ± 0.06 *
Amplitude of spontaneous APs (mV)	45.2 ± 3.5	47.8 ± 4	53.8 ± 3.9	48.8 ± 2.3	28.57 ± 6.5	42.8 ± 3	46.5 ± 2.5	43.3 ± 2
Cells with miniature PSCs (%)	88.8 ± 6.6	87.5 ± 7.2	40.7 ± 12.1	74 ± 8.5	56.3 ± 21.4	73.3 ± 17.6	76.7 ± 5.1	67.5 ± 9.6
Miniature PSC frequencies (Hz)	0.59 ± 0.08	0.45 ± 0.05	0.88 ± 0.3	0.6 ± 0.07	0.17 ± 0.02	0.43 ± 0.08	0.31 ± 0.03	0.29 ± 0.03 ***
Miniature PSC amplitudes (pA)	42.4 ± 3.3	29.4 ± 1.6	38.9 ± 2.8	37.4 ± 1.8	22.7 ± 2.7	28.4 ± 2.5	37.9 ± 2.9	29.5 ± 1.7 ***

Abbreviations: Voltage-gated sodium (I_Na_), potassium currents (I_K_), action potentials (APs), afterhyperpolarization (AHP), postsynaptic currents (PSC). Data are given as means ± SEM (* p < 0.05, *** p < 0.001, nonparametric Mann–Whitney test). Bold values indicate significant results.

## Data Availability

Data contained within the article or supplementary material are available from the corresponding author upon reasonable request.

## Supplementary Materials

The following supporting information can be downloaded at: https://www.mdpi.com/article/10.3390/cells12020223/s1, Figure S1: Quantification of immunocytochemistry stainings of mature MSNs; Figure S2: Spontaneous calcium (Ca^2+^) signaling of MSNs derived from patients with MSA-P and healthy controls; Figure S3: Resting membrane potential, membrane capacitance, and input resistance measured by voltage-gated patch-clamp recordings; Figure S4: Characteristics of evoked action potentials of MSNs; Figure S5: Properties of spontaneous action potentials of MSNs; Figure S6: Characteristics of miniature postsynaptic currents (mPSCs) of MSNs. Table S1: List of primary antibodies; Table S2: List of secondary antibodies; Table S3: List of oligosequences used for quantitative RT-PCR analysis.
