# Supplementary material for: iPSC-Derived Striatal Medium Spiny Neurons from Patients with Multiple System Atrophy Show Hypoexcitability and Elevated α-Synuclein Release
Source: Cells. 2023 Jan 4;12(2):223. doi: 10.3390/cells12020223 (PMC9856678; doi:10.3390/cells12020223)
Supplement: Supplementary file 1 [file cells-12-00223-s001.zip › cells-2124952-supplementary.pdf]

Supplemental information

Tables

Supplementary Table S1. List of primary antibodies used in this study

Antibody	Species	Isotype	Dilution	Cat.Nr.	Source
**α-synuclein**	mouse	IgG1	1:500	sc-12767	Santa Cruz Biotechnology, Dallas, TX, USA
**α-synuclein**	rabbit	IgG (H+L)	1:1000	2642S	Cell Signaling Technology, Danvers, MA, USA
**β-actin**	rabbit	IgG (H+L)	1:1000	5125S	Cell Signaling Technology, Danvers, MA, USA
**CTIP2**	rat	IgG2a	1:300	ab18465	Abcam, Cambridge, MA, USA
**GABA**	mouse	IgG1	1:500	A0310	Sigma-Aldrich, St. Louis, MO, USA
**GABA**	rabbit	IgG (H+L)	1:1000	A2052	Sigma-Aldrich, St. Louis, MO, USA
**GAPDH**	mouse	IgG1	1:1000	sc-32233	Santa Cruz Biotechnology, Dallas, TX, USA
**GATA-4**	rabbit	IgG (H+L)	1:400	MA5-32678	Thermo Fisher Scientific, Waltham, MA, USA
**Nestin**	mouse	IgG1	1:300	MAB1259	R&D Systems, Minnesota, MN, USA
**OCT4**	rabbit	IgG (H+L)	1:400	2750S	Cell Signaling Technology, Danvers, MA, USA
**PAX6**	mouse	IgG1	1:400	ab78545	Abcam, Cambridge, MA, USA
**SOX2**	rabbit		1:500	3579P	Cell Signaling Technology, Danvers, MA, USA
**TRA-1-60**	mouse	IgM	1:400	ab16288	Abcam, Cambridge, MA, USA
**TUJ1**	mouse	IgG2a	1:1000	ab78078	Abcam, Cambridge, MA, USA
**TUJ1**	rabbit	IgG (H+L)	1:1000	ab18207	Abcam, Cambridge, MA, USA
**Vimentin**	mouse	IgG1	1:400	ab8978	Abcam, Cambridge, MA, USA
**DAPI**					Thermo Fisher Scientific, Waltham, MA, USA

Abbreviations: COUP TF1-interacting protein 2 (CTIP2), γ-aminobutyric acid (GABA), GATA binding protein 4 (GATA-4), octamer-binding transcription factor 4 (OCT4), paired box protein Pax-6 (PAX6), SRY (sex determining region Y)-box 2 (SOX2), Podocalyxin (TRA-1-60), β-tubulin III (TUJ1), 4,6-diamidino-2-phenylindole (DAPI).

Supplementary Table S2. List of secondary antibodies used in this study

Target species	Host species	Target antigen	AlexaFluor	Dilution	Cat.Nr.	Source
**mouse**		IgG1	555		A21127	
**mouse**		IgG2a	488		A21131	
**mouse**		IgM	488		A21042	
**rabbit**		IgG (H+L)	488		A11034	Thermo Fisher
	goat			1:1000		Scientific, Waltham,
**rabbit**		IgG (H+L)	555		A21428	MA, USA
**rat**		IgG (H+L)	555		A21434	
**mouse**	horse	IgG (H+L)	na (Dot Blot)	1:2500	PI-2000-1	Vector Laboratories,
**rabbit**	goat	IgG (H+L)		1:5000	PI-1000-1	Burlingame, CA, USA

Supplementary Table S3. List of oligosequences used for quantitative RT-PCR analysis

Target	Primer sequence (5′ ⟶ 3′)
iPSC characterization	
**c-Myc**	fwd: CCAGCAGCGACTCTGAGGA
	rev: GAGCCTGCCTCTTTTCCACAG
**Ki67**	fwd: AAGTACATGTGCCTGCTCGACCC
	rev: TGTGACATGTGCTTGTCAACTGCG
**Nanog**	fwd: CACCTATGCCTGTGATTTGTGGGC
	rev: AGAAGTGGGTTGTTTGCCTTTGGG
**Oct4**	fwd: GGAAGGAATTGGGAACACAAAGG
	rev: AACTTCACCTTCCCTCCAACCA
MSN characterization	
**CTIP2**	fwd: CTCCGAGCTCAGGAAAGTGTC
	rev: TCATCTTTACCTGCAATGTTCTCC
**FOXP1**	fwd: CCACGTGGAAGAATGCAGTGCG
	rev: GCATTGAGAGGTGTGCAGTAGGC
**GAD67**	fwd: AGATCAACAAATGCCTGGAACTGGC
	rev: GAGCCACCTTGTGTAGCTTTTCCC
**MAP2**	fwd: CAGGCAAAGGACAAAGTCTCTGACG
	rev: CGCCGAGGAGGGAGAATGGAGG
**SST**	fwd: GAGATCTGCTAACTCAAACCCGGC
	rev: TCGCTGAAGACTTGGAGGATTAGGG
**α-Syn**	fwd: AAGAGGGTGTTCTCTATGTAGGC
	rev: GCTCCTCCAACATTTGTCACTT
$GABA_A$ receptor subunit	

**α1**	fwd: TGCAGCTTGGAGACAGGATT<br>rev: TGAACCATCTTCCCCCTCTT
**α2**	fwd: AGAGGATGGACTTGGGATGG<br>rev: AAGATTCGGGGCATAATTGG
**α3**	fwd: CACAAGTGTCGTTCTGGCTCA<br>rev: TGGCACTGATACTCAAGGTGGT
**α4**	fwd: TCCGGTTTTCATGCAAAGGT<br>rev: CTTCATTAAGGATAAGCCAGTGGAA
**α5**	fwd: GGTGTCCTTTTGGCTGAACC<br>rev: GCCACTTTGGGCAGAGAGTT
**α6**	fwd: TTTCCCAGGTGTCTTTCTGGA<br>rev: GGCACTGATGCTCAAAGTGG
**β1**	fwd: ATGCATCTGCAGCCAGAGTC<br>rev: AGGGATCTTTGGCAGGGTCT
**β2**	fwd: CCCAAACCAAATGTCACTGC<br>rev: TGGAACTGTCAACTTGCTTCAAA
**β3**	fwd: ATTGAAAGGCGCCATGTTTT<br>rev: GGGTTGGTCCTAGGGAGAGG
**γ1**	fwd: GGAGATGGGGGATGATAGGC<br>rev: ATCCCTTCCACCCAACACAC
**γ2**	fwd: TTGTCGAACAGGAGCTTGGA<br>rev: GAAGGCAGTGGGGAAGAAGA
**γ3**	fwd: AACCAACCACCACGAAGAAGA<br>rev: CCTCATGTCCAGGAGGGAAT
**δ**	fwd: GTCTTTGCTCTGCAGGATCG<br>rev: CCAGGCCAAGGCTTTATTTC
$GABA_B$ receptor subunit	
**GABBR1**	fwd: AGATGACTGAGGCGGTGGA<br>rev: TTCAGCCGCTTGGTTAGTTTC
**GABBR2**	fwd: GAGCAGATCCGCAACGAGTCAC<br>rev: GACAGACGCCTCCAAACACCATC
ATP-regulated potassium (K_{ATP}) channels	
**ABCC8**	fwd: AACAACGGCTGCTTTGTGGACG<br>rev: CAGGTTGTGCCCAGGGAAATGAAG
**ABCC9**	fwd: TTGAAGCAACCAGAAGTAGGAACAGG<br>rev: GGCTGAAGAGAACAGGCATCTGTG
voltage-gated Ca^{2+}-channel subunit	
**Ca_v 1.2 (L-type)**	fwd: CATTTGACGCCTTGATTGTTGTGGG<br>rev: GTATGTTCAGCTGGGTTTACCTCGG
**Ca_v 1.3 (L-type)**	fwd: CGGACCCCGTCCTCGAAGGA<br>rev: CCTACGCGGATCGGGTTGGT
**Ca_v 2.1 (P-type)**	fwd: CCAGAAACTTGCCCTACAGAAAGCC<br>rev: CGGGTCCATTTCGTTATACAGGGC
**Ca_v 2.2 (N-type)**	fwd: TGCTGTTCAGGAGCGCCACG<br>rev: CGGTGGCATTGGCCTGCTCA

**Cav 2.3 (R-type)**	fwd: GTGGCCCTGGGGTTCATCTTCCATA<br>rev: CAGGATGCCACTGAGGACCACGA
**Cav 3.1 (T-type)**	fwd: TCAGCCTCCCCCTGAGCGTG<br>rev: TTCTGCAGGACCGCATGCCG
**Cav 3.2 (T-type)**	fwd: GTCACTCTGCTGCTGGATACGC<br>rev: TCAGGTTGTTGTTCCTGACAAAGGC
**Cav 3.3 (T-type)**	fwd: ATCGACTACACCCTGTGCTTCCG<br>rev: GACGTAGTCGAAGAGTTTGTGGGC
reference genes	
**B2M**	fwd: TGCCTGCCGTGTGAACCATGT<br>rev: TGCGGAATCTTCAAACCTCCATGA
**GAPDH**	fwd: AGCCACATCGCTCAGACACCAT<br>rev: CAGGCGCCCAATACGACCAAAT
**β-actin**	fwd: CATGTACGTTGCTATCCAGGC<br>rev: CTCCTTAATGTCACGCACGAT

Figures

Supplementary Figure S1

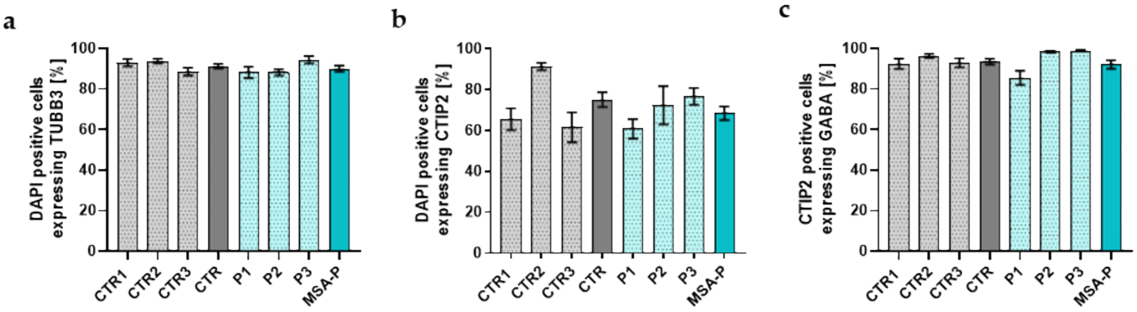

Supplementary Figure S1: Quantification of immunocytochemistry stainings of the mature MSNs at day 70 (± 3 days) of differentiation, confirming the expression of (a) TUBB3 and (b) CTIP2 in 88-95% and 61-95% of all cells analyzed, respectively, as well as (c) GABA in 86-99% of the CTIP2-positive cells. Each cell line used in this study was analyzed separately and is shown separately.

Supplementary Figure S2

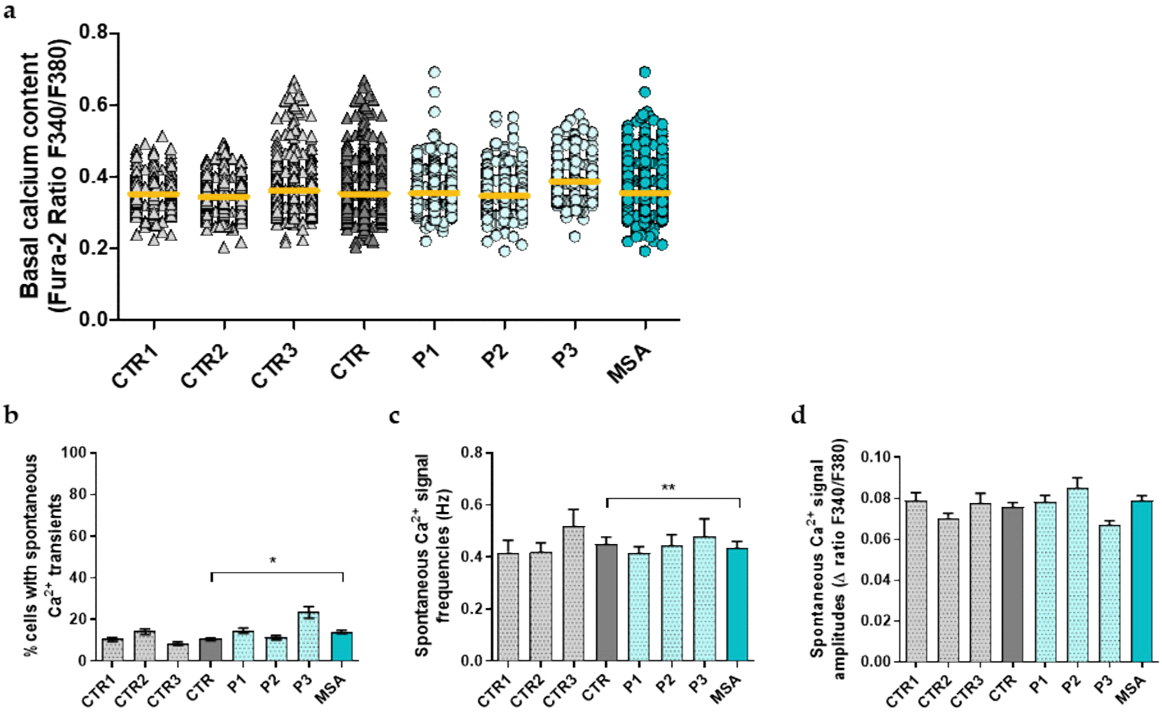

Supplementary Figure S2: Spontaneous calcium (Ca^{2+}) signaling of the MSNs derived from the patients with MSA-P and the healthy controls. Intracellular Ca^{2+} transients are presented as ratios of the fluorescence signals obtained at 340 and 380 nm (F_{340}/F_{380}). (**a**) Basal intracellular Ca^{2+} levels of all cell lines. (**b**) Percentage of cells exhibiting spontaneous Ca^{2+} transients, (p = 0.0251, nonparametric Mann–Whitney test) (**c**) frequency (p = 0.0047, nonparametric Mann–Whitney test) and (**d**) amplitudes of these transients. Each cell line was analyzed separately and is shown separately. Data are presented as means ± SEM.

Supplementary Figure S3

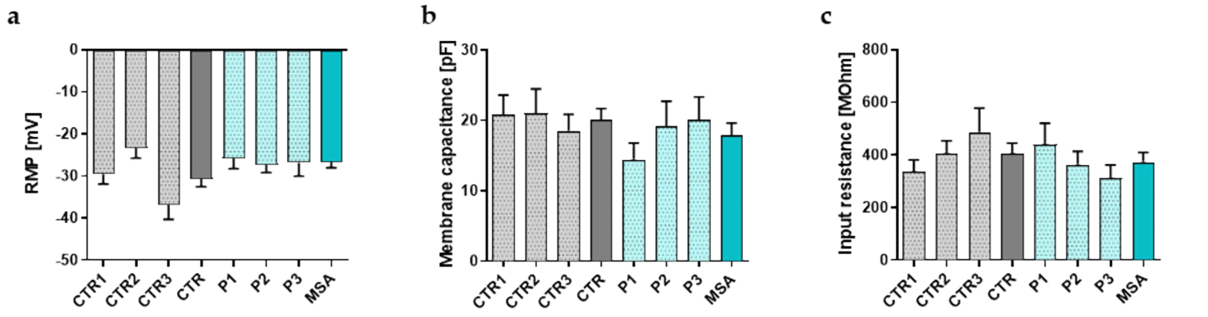

Supplementary Figure S3: Resting membrane potential (**a**), membrane capacitance (**b**), and input resistance (**c**) of each cell line measured by voltage-gated patch-clamp recordings. Data are presented as means ± SEM.

Supplementary Figure S4

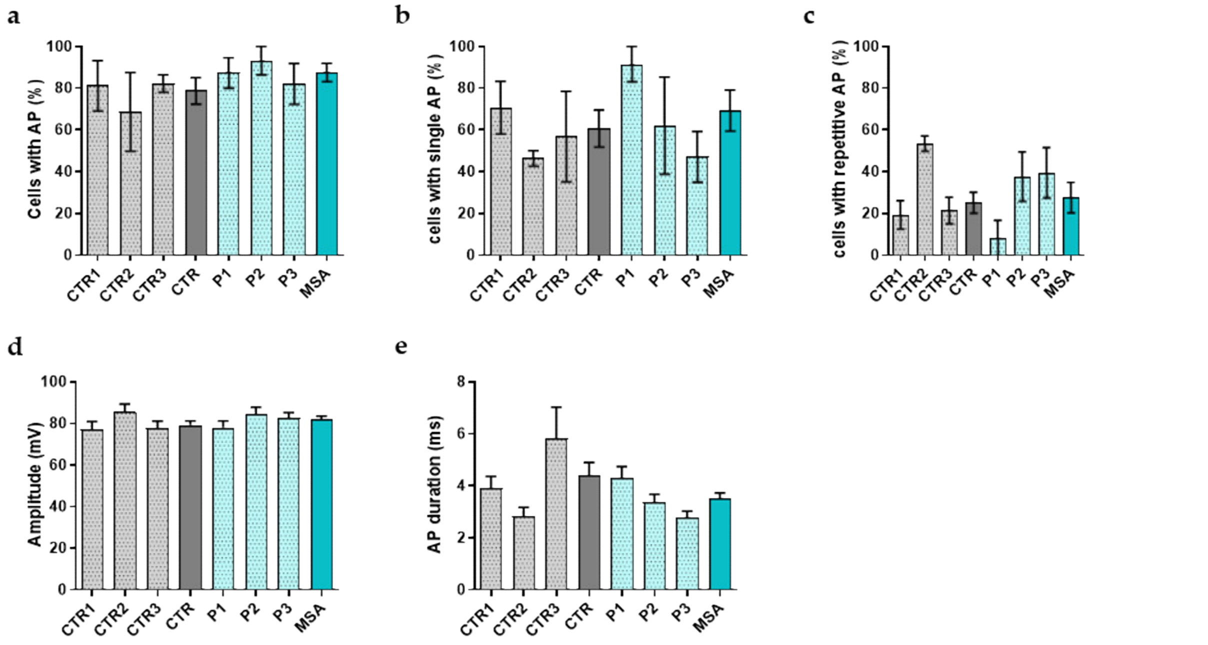

Supplementary Figure S4: (**a**) Percentage of cells able to spike action potentials (APs) evoked by depolarizing current pulses, (**b**) percentage of cells that produced a single evoked AP, and (**c**) percentage of cells with repetitive firing. (**d**) Amplitude and (**e**) duration of evoked APs measured by voltage-gated patch-clamp recordings for each cell line. Data are presented as means ± SEM.

Supplementary Figure S5

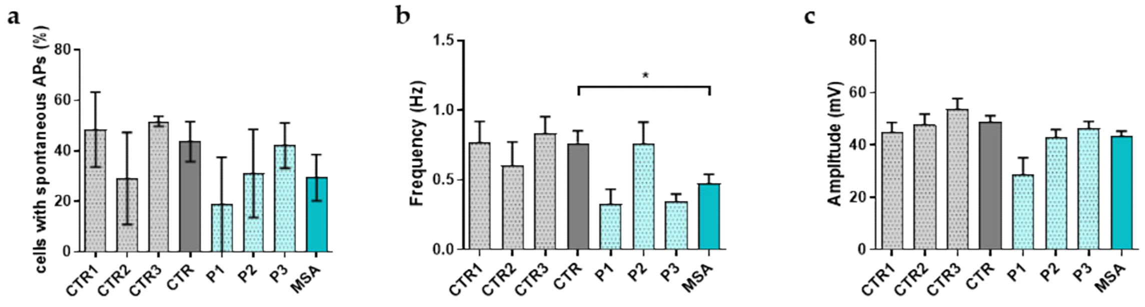

Supplementary Figure S5: Properties of spontaneous APs in the MSNs from the MSA-P patients and the healthy controls. (**a**) Percentage of neurons with spontaneous APs, (**b**) frequency ($p = 0.0291$, nonparametric Mann–Whitney test), and (c) amplitudes of spontaneous APs in all cell lines used in this study. Data are presented as means ± SEM.

Supplementary Figure S6

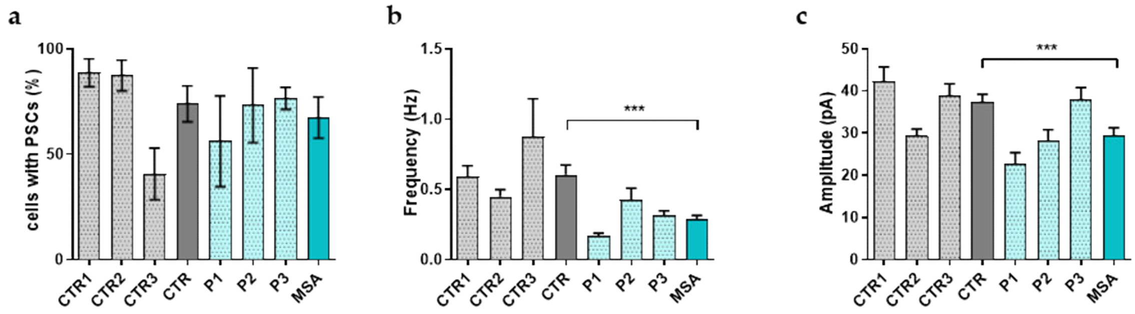

Supplementary Figure S6: Characteristics of miniature postsynaptic currents (mPSCs) in the MSNs from the MSA-P patients and the healthy controls. (**a**) Percentage of neurons showing mPSCs, (**b**) frequency ($p = 0.0007$, non-parametric Mann–Whitney test), and (c) amplitudes of mPSCs ($p < 0.0001$, nonparametric Mann–Whitney test) in all cell lines used in this study. Data are presented as means ± SEM.
